# Music in the Treatment of Insanity

**Published:** 1886-12

**Authors:** 


### Medical News.

Music in the Treatment of Insanity.—That music has sedative and stimulating powers is a fact which has been known since the days when Orpheus quelled the savage Cerberus and David quieted the raving Saul. The only wonder is that, in these days of hydropathy, homœopathy, mind and prayer cures, no school of musicopathy has arisen. Soft, sad strains might be employed to quiet the maniacal, merry allegretto music for the cure of melancholia, while Wagner might frighten the demented so that he would begin to think again. Dr. Talcott, Superintendent of the Middletown, N. Y., State Asylum for the Insane, has evidently thought of these things and introduced music into all of the wards of his institution with excellent results judging from his last annual report from which the following is extracted: "It is said, that, before Moses dwelt upon the banks of the Nile, the Egyptians erected temples and altars for the treatment of the insane; and, among the most notable measures for the accomplishment of the cure of lunatics, music took an exalted rank. There can be no doubt that music exercises a potent influence in producing calm and restfulness in minds which are disturbed by cerebral disease. Musical instruments have been provided in nearly every ward, and the results have been most favorable. Even turbulent patients will subside when the pleasures of music are afforded to them. One of the most effective attendants we ever had upon our disturbed wards was a good musician. After his work was done, he would sit down among his patients, and play upon the violin. Immediately the most excited persons in the ward would group themselves about him, and listen with profound attention so long as he continued to play for them. Where good music can be provided for the turbulent insane, there exists but little necessity for restraint of a physical nature."

